# Listening to the HysterSisters: A Retrospective Keyword Frequency Analysis of Conversations About Hysterectomy Recovery

**DOI:** 10.2196/10728

**Published:** 2019-09-26

**Authors:** Arpit Dave, Johnny Yi, Andy Boothe, Helene Brashear, Jeffrey Byrne, Yash Gad

**Affiliations:** 1 Mayo Clinic Arizona Department of Gynecology Phoenix, AZ United States; 2 W2O Group Austin, TX United States

**Keywords:** hysterectomy, gynecology, social media, perceived recovery

## Abstract

**Background:**

In the postoperative period, individual patient experiences vary widely and are based on a diverse set of input variables influenced by all stakeholders in and throughout the surgical process. Although clinical research has primarily focused on clinical and administrative datasets to characterize the postoperative recovery experience, there is increasing interest in patient-reported outcome measures (PROMs). The growth of online communities in which patients themselves participate provides a venue to study PROMs directly. One such forum-based community is HysterSisters, dedicated to helping individuals through the experience of hysterectomy, a major surgery which removes the uterus. The surgery can be performed by a variety of methods such as minimally invasive approaches or the traditional abdominal approach using a larger incision. The community offers support for “medical and emotional issues [...] from diagnosis, to treatment, to recovery.” Users can specify when and what type of hysterectomy they underwent. They can discuss their shared experience of hysterectomy and provide, among other interactions, feedback, reassurance, sympathy, or advice, thus providing a unique view into conversations surrounding the hysterectomy experience.

**Objective:**

We aimed to characterize conversations about hysterectomy recovery as experienced by users of the HysterSisters online community.

**Methods:**

A retrospective keyword frequency analysis of the HysterSisters Hysterectomy Recovery forum was performed.

**Results:**

Within the Hysterectomy Recovery forum, 33,311 unique users declared their hysterectomy date and type and posted during the first 12 weeks postsurgery. A taxonomy of 8 primary symptom groups was created using a seed list of keywords generated from a term frequency analysis of these threads. Pain and bleeding were the two most mentioned symptom groups and account for almost half of all symptom mentions (19,965/40,127). For symptoms categories such as pain and hormones and emotions, there was no difference in the proportion of users mentioning related keywords, regardless of the type of hysterectomy, whereas bleeding-related or intimacy-related keywords were mentioned more frequently by users undergoing certain minimally invasive approaches when compared with those undergoing abdominal hysterectomy. Temporal patterns in symptom mentions were noted as well. The majority of all posting activity occurred in the first 3 weeks. Across all keyword groups, individuals reporting minimally invasive procedures ceased forum use of these keywords significantly earlier than those reporting abdominal hysterectomy. Peaks in conversation volume surrounding particular symptom categories were also identified at 1, 3, and 6 weeks postoperatively.

**Conclusions:**

The HysterSisters Hysterectomy Recovery forum and other such forums centered on users’ health care experience can provide novel actionable insights that can improve patient-centered care during the postoperative period. This study adds another dimension to the utility of social media analytics by demonstrating that measurement of post volumes and distribution of symptom mentions over time reveal key opportunities for beneficial symptom-specific patient engagement.

## Introduction

In the postoperative period, individual patient experiences vary widely and are based on a diverse set of input variables influenced by all stakeholders in and throughout the surgical process [1-3]. Postoperative recovery has been defined as “a dynamic process in an endeavor to continue with everyday life” wherein “individuals strive and struggle to gain independence and return to everyday life” [4]. A total of 4 dimensions of the postoperative recovery process have been described: physiological, psychological, social, and habitual [1]. Although clinical research has focused heavily on the physiological aspect through analysis of clinical and administrative datasets, there is increasing interest in patient-reported outcome measures (PROM), where patients report directly on their own recovery process or experience [5-8]. Standardized validated instruments such as the Quality of Recovery-40 have been developed to elicit this direct information [9]. These PROMs can then ostensibly be used to improve quality of care.

The growth of online communities in which patients themselves participate provides an alternate venue to study PROMs. The authors leveraged this participation to study PROMs in online discussions about postoperative recovery initiated by people who reported undergoing hysterectomy. Hysterectomy is a surgery to remove the uterus and is done for a variety of reasons including leiomyoma (benign smooth muscle tumor), abnormal uterine bleeding, or gynecologic malignancies and can be performed through a variety of surgical approaches [10-13].

The site of the discussions was HysterSisters, an online community dedicated to “issues surrounding the hysterectomy experience […] from diagnosis, to treatment, to recovery” [14]. An active forum dedicated to hysterectomy recovery contains posts from users dating back over 10 years and includes detailed information including the date and type of hysterectomy and conversations about their actual experience. A survey of the HysterSisters community showed that top motivations for posting were obtaining information (87%), experience sharing (76%), and offering advice or information (70%) [15].

Previous work on HysterSisters administered a Likert-based satisfaction survey to a self-selected population of forum participants [16]. The survey covered a variety of general postoperative recovery domains including overall hysterectomy results, time to return to normal activity, pain and discomfort, and others. Here, we build on this work by performing a linguistic analysis of the posts from the population of site users who participated in a posthysterectomy recovery forum.

There are numerous techniques with which to approach the content analysis of online forum posts [17,18]. The choice of tool is dependent on the information sought. Tools such as Linguistic Inquiry and Word Count use preset dictionaries to assist in tasks such as sentiment analysis, whereas use of a topic modeling strategy such as latent Dirichlet allocation or a simpler term frequency analysis can identify *topics* of conversation within a *bag-of-words* corpus of forum text [19,20].

This study attempted to characterize more completely the recovery experience of individuals in the HysterSisters community by studying the subject headings of users’ own publicly available posts to *listen* to conversations outside the provider’s office. Therefore, the objective was to identify conversation patterns in forum data to provide actionable insights into the surgical recovery experience.

## Methods

### Study Design

In accordance with the Code of Federal Regulations 45 CFR 46, the Mayo Clinic Institutional Review Board (IRB) deemed this study does not require IRB review on the basis that publicly accessible contributions to the HysterSisters forums do not constitute private behavior. No registration or login was required to read posts.

We performed a retrospective mixed methods analysis of forum posts that leveraged both structured and free-text user inputs. Qualitative assessments included development of a *symptom* taxonomy of forum topic keywords that encompass the 4 dimensions of recovery as described previously. Although each keyword may not represent a medical or pathologic symptom and should not be equated with a patient complaint, we use the word in its broader context as an indicator of an element of the general recovery experience. Quantitative investigations included keyword frequency analyses and survival analyses of these keywords, each of which is described below.

### Data Collection

The HysterSisters website provided users with the option to enter structured data including type of hysterectomy, ovarian status, and exact date of procedure [8]. The forum’s taxonomy of hysterectomy types was categorized for analysis into *treatment groups* by surgical approach (Textbox 1). As there are many approaches to hysterectomy, the question of which approach is superior is of interest, as are reasons a surgeon or patient might choose one approach over another. The American College of Obstetrics and Gynecology considers both the vaginal hysterectomy (VH) and laparoscopic hysterectomy (LH) to be *minimally invasive*, with well-described benefits over the AH approach, and a recent Cochrane systematic review also demonstrated favorable clinical outcomes for both VH and LH when compared with AH [21,22]. We chose a similar organizational scheme to study the question of hysterectomy recovery experience from the forum user’s own words.

**HysterSisters website taxonomy for hysterectomy (available hysterectomy types categorized into treatment groups by surgical approach).**

**Abdominal**

1. Total abdominal (TAH)
2. Supracervical abdominal (SAH)
3. Either, not specified (TAH/SAH)

**Vaginal (minimally invasive surgery)**

1. Total vaginal (TVH)
2. Laparoscopic-assisted vaginal (LAVH)

**Laparoscopic (minimally invasive surgery)**

1. Total laparoscopic (TLH)
2. Laparoscopic supracervical (LSH)
3. da Vinci robotic laparoscopic (DVH)
4. Single-incision laparoscopic (SILS or laparoendoscopic single-site surgery [LESS])

As seen in these previous studies, LAVH is difficult to categorize as the procedure contains elements of both total vaginal hysterectomy and total laparoscopic hysterectomy procedures. In this instance, we opted to include with the vaginal group as traditionally, most critical portions of the procedure are performed through the vagina. Single-incision laparoscopic surgery (SILS), also known as laparoendoscopic single-site surgery (LESS) represented fewer than 100 individuals and was excluded from the analysis.

The *Hysterectomy Recovery* (*posthysterectomy*) board was selected to focus specifically on individuals who were posthysterectomy and in early recovery. This board was constrained to posts from users who had undergone hysterectomy who were initiating a conversation by selecting only the subject heading from the first post in each thread. The initial post body and subsequent thread replies were excluded to simplify computation and limit the analysis to the original posting users. Posts from users who did not declare a hysterectomy type and date were excluded because of being unable to reliably determine if and when these users underwent hysterectomy and what type. Posts preceding the individuals’ hysterectomy date or more than 12 weeks afterward were also excluded. In the clinical setting, the traditional postoperative healing period for hysterectomy is thought to last 6 weeks, after which patients are typically discharged to routine annual follow-up. However, the research by Vonk Noordegraaf et al has shown that median time to return to work may be upward of 8 weeks [23]. We sought to determine if users continued to engage beyond this time frame, given that (1) anecdotal evidence suggests that patients experience *subclinical* complications of surgery (eg, bloating, discomfort, and hormonal symptoms) beyond 6 weeks, and (2) a major complication of hysterectomy (dehiscence of the vaginal cuff) has been demonstrated to have a median time to occurrence as long as 11 weeks [24]. We therefore chose to look out 12 weeks from the reported date of hysterectomy. We did not exclude any users based on reporting of whether ovaries were removed at the time of hysterectomy. Although posts in the board were public, data collection preserved anonymity by processing forum text and metadata without the username.

### Symptom Keyword Frequency Analysis

Term frequency analysis is an analytical technique that characterizes the differences between 2 text corpora by comparing the relative frequencies at which n-grams appear in each corpus [25,26]. N-grams are a contiguous sequence of n words, where n is an integer. This n-gram analysis used a log-likelihood approach to term frequency analysis because it offers a test for significance [27]. To gain understanding of how users in different treatment groups discussed the same symptom differently, 1 corpus each was constructed from the subjects of the posts mentioning the given symptom for the 2 treatment groups to compare, and then term frequency analysis was applied to compare the 2 corpora. For this analysis, the abdominal treatment group subject headers were used as the base corpus, against which each minimally invasive surgery group (laparoscopic and vaginal) was individually compared. The rationale for this comparison emerges from the consensus in clinical gynecology that the minimally invasive treatments are preferable to traditional AH because of less surgical risk and faster recovery, as we sought to identify if these patterns emerged in online conversations as well [21,22]. This analysis yielded a list of n-grams used more by each treatment group when mentioning each symptom versus the abdominal treatment group; these lists prioritized and motivated any manual, qualitative review of matching posts. All comparisons used equality to the abdominal treatment group’s mention frequency as the null hypothesis to allow comparison of the set of minimally invasive treatments with the traditional treatment.

### Symptom Taxonomy

To translate agnostic forum text into clinically meaningful information, a symptom keyword taxonomy was developed using a subjective, iterative, collaborative process between the medical and computational researchers. The initial keywords were identified using an n-gram (n=1, 2, and 3) frequency analysis of all posts in the *Hysterectomy Recovery* board, which revealed a *seed* list of commonly reported words used to describe symptoms. The list was then expanded by alternately searching by keywords and inspecting text that was both included and excluded by search queries. Keyword searches were used to pull subject headers containing the keyword and then new keywords were added to the list after manually examining the conversations beneath that header. This process continued until the keyword searches maintained a consistent conversation indexing. Individual keywords were chosen to prioritize specificity over sensitivity; sensitivity was maximized by including many keywords.

The final keyword symptom groups emerged as *pain*, *sleep and fatigue*, *hormones and emotions*, *digestion*, *swelling*, *bleeding*, *urination*, *intimacy*, *odd sensations*, *drugs*, *fever and infection*, and *family*. The set of all words for the keyword symptom groups included technically accurate terms (*gastritis*), proper English words (eg, *itches* and *burns*), slangs (eg, *weepies* and *swellybelly*), and common typographical and spelling errors (eg, *achey* and *vomitting*). Multimedia Appendix 1 shows the keyword symptom groups and all included words, organized alphabetically, and Multimedia Appendix 2 shows the same, sorted by number of mentions in descending order.

### Symptom Keyword Mention Frequency Analysis

The symptom mention frequency analysis compared the number of users who mentioned each symptom broken out by treatment group. The subject headers were tagged for symptom mentions by searching tokenized post subjects for the corresponding symptom keywords from the taxonomy. Responses were aggregated by user to compile the list of symptoms each user mentioned during the 12-week postoperative recovery period. A chi-square test for homogeneity was used to compare the mention frequencies of each symptom among individuals in each hysterectomy group with the abdominal group.

### Symptom Keyword Mention Survival Analysis

To compare whether individuals undergoing minimally invasive treatments stopped discussion of symptoms on the forum earlier than their counterparts undergoing AH, the same set of tagged subject headers was grouped by user and sorted chronologically to determine each user’s latest mention of each symptom. Those who did not mention a symptom were excluded from the survival analysis of that symptom only; in the event that more than 1 symptom category was mentioned by a user, each symptom category was considered separately. A log-rank test was used to compare the final mentions of a symptom among each treatment group. All comparisons used equality to the abdominal treatment group’s corresponding symptom survival curve as the null hypothesis. Mean interquartile difference between survival curve pairs were calculated to quantify which group ceased to mention symptom keywords earlier.

### Software

All data processing and analysis were done using free, open-source libraries written in Python (Python Software Foundation). Data processing and aggregation were performed using the *pandas* library, text processing with the *nltk* library, and statistical analysis using the *scipy* library.

## Results

### Summary Statistics

**Figure 1:**
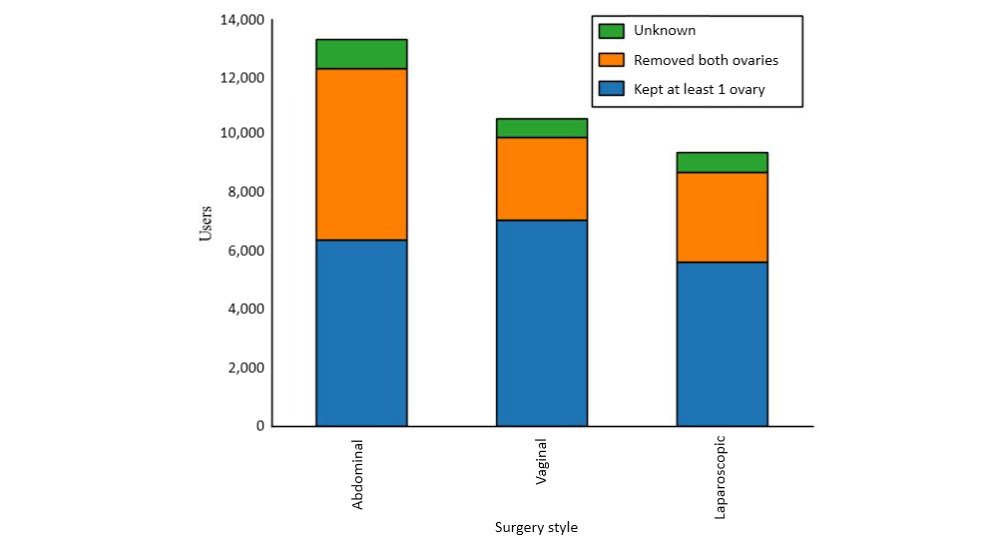
Ovary status by surgical approach. Total number of procedures reported by HysterSisters patients mentioning at least one symptom included in the taxonomy, stratified by surgical approach. Each procedure is broken down by ovary status. Unknown indicates patients did not provide ovary status data.

There were 33,311 unique users in the Hysterectomy Recovery forum, making at least one mention of a symptom in the taxonomy. Among these contributors, the procedure distribution is as follows: abdominal=13,306/33,311 (39.94%), vaginal=10,589/33,311 (31.79%), and laparoscopic=9416/33,311 (28.27%). Among users who provided ovary status data, there were more who kept at least one ovary (18,645/33,311, 55.97% of all users) than who had both removed (12,313/33,311, 36.96%); some did not specify their ovary status (2,353/33,311, 7.06%). Ovary status by treatment group is shown in Figure 1.

### Conversation Volume

Site users with completed profiles created a total of 80,704 top-level posts during the first 12 weeks of their respective recoveries. The subjects of 42.43% (34,242/80,704) of these posts mentioned at least one symptom as defined by the symptom taxonomy; the remaining 57.57% (46,462/80,704) mentioned none.

**Figure 2:**
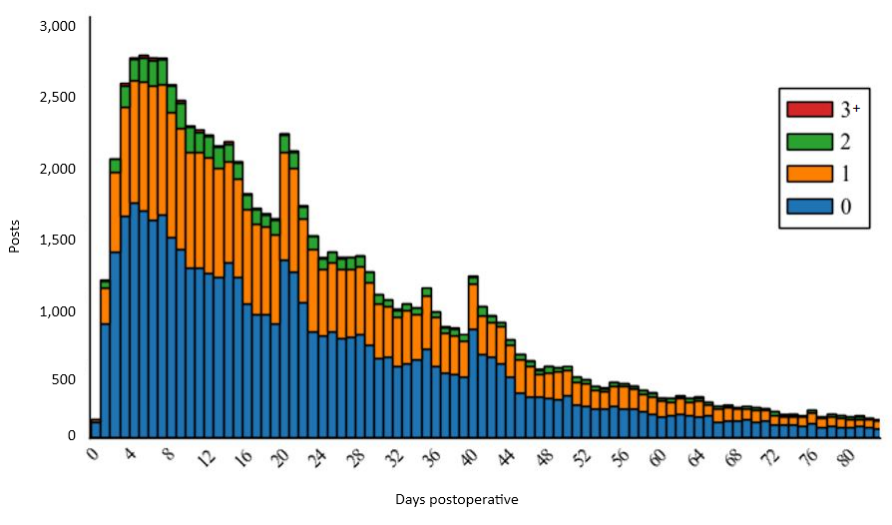
Post volume by days postoperative. Bars indicate total number of posts created by HysterSisters patients, grouped by the number of whole days postoperation the post was created. Bars are broken down by number of symptoms each post subject mentions according to the symptom taxonomy.

Posting behavior was heavily skewed, with most posts (42,910/80,704, 53.17%) happening within the first 3 weeks of the 12-week recovery period being studied. Figure 2 shows a histogram of posts, segmented by days postoperation and stratified by symptom mention count. The median post was made during day 19 (μ=day 23.80 and σ=18.56 days). The median posts per contributor was 1 post (μ=2.42 posts and σ=3.10 posts).

### Symptom Mention Volume

Multimedia Appendix 3 shows a bar chart of users who mention each symptom. The top 3 symptoms by volume of mentions are pain, bleeding, and *hormones and emotions* for both the aggregate conversation and each of the conversations by procedure.

### Sorting by Relevance

**Figure 3:**
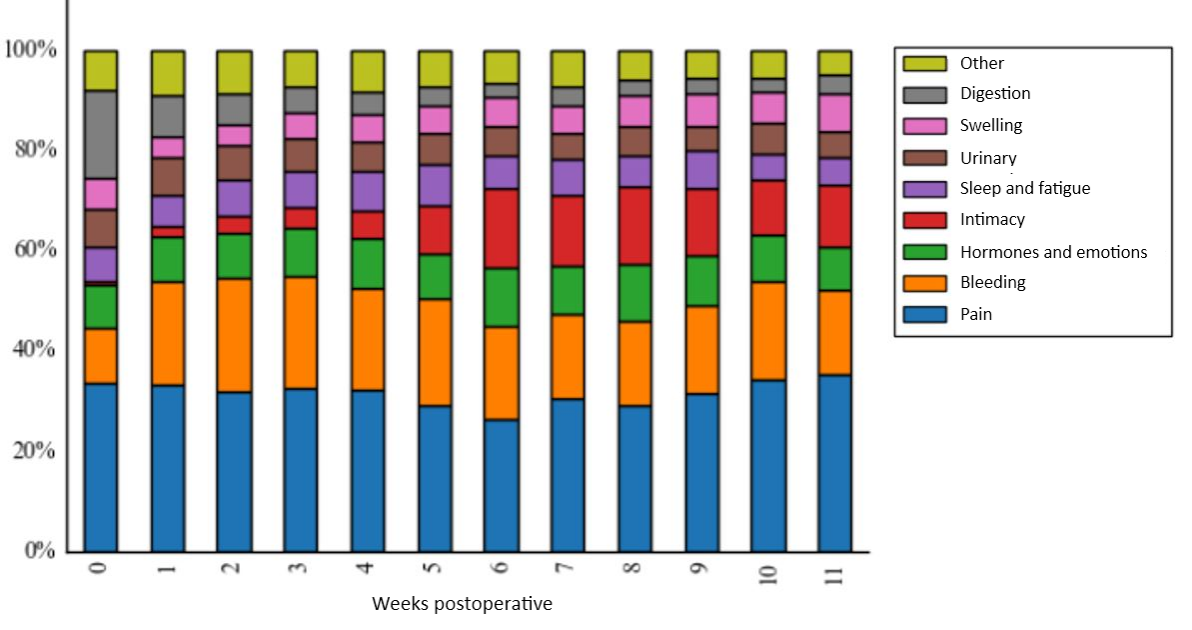
Symptom mention distribution over time, by week. Distribution of all symptom mentions, grouped by number of whole weeks postoperation.

The 34,242 subject headers, which mention at least one symptom contain 40,127 total symptom mentions. The symptoms mentioned the most were pain (12,474/34,244, 36.43% of subject headers that mention at least 1 symptom) and bleeding (7,491/34,242, 21.88%); together, these symptoms account for half of all symptom mentions. Relevant posting behavior follows overall posting volume very closely. Proportional symptom mentions per unit time remain generally flat throughout the first 12-weeks of recovery with few important exceptions (Figure 3).

### User Symptom Keyword Mention Frequency Analysis

There is a significant difference between the number of users who mention a symptom at any point during recovery for a given treatment group versus the abdominal group for some symptoms. For example, users attesting to VH mentioned urinary and intimacy keywords proportionally more. Users having had an LH mentioned bleeding proportionally more. Both mentioned swelling and sleep and fatigue-related keywords less. There were no differences in the frequency of mentions of pain and hormone and emotion keywords. Table 1 shows the absolute percentage difference in mentions for each treatment group and symptom permutation versus the abdominal surgical group.

**Table 1:** Absolute percentage difference for mentions of a given symptom by procedure compared with the abdominal group.

Symptom keyword	Laparoscopic (%)	Vaginal (%)
Family	−0.05^a^	0.38^b^
Drugs	−0.81^a^	−0.60^a^
Urinary	−0.50^a^	2.29^b,c^
Hormones and emotions	−0.49^a^	0.28^b^
Intimacy	−0.25^a^	2.38^b,c^
Sleep and Fatigue	−2.22^a,d^	−1.69^a,e^
Swelling	−2.94^a,d^	−3.04^a,c^
Pain	0.21^b^	0.98^b^
Fever and infection	−0.42^a^	0.39^b^
Digestion	1.55^b^	0.41^b^
Odd sensations	−0.43^a^	−0.96^a^
Bleeding	3.03^a,d^	−1.46^b^

^a^Values indicate the abdominal cohort mentions the symptom more.

^b^Values indicate the abdominal cohort mentions the symptoms less.

^c^*P*<.001.

^d^*P*<.01.

^e^*P*<.05

### User Symptom Keyword Mention Survival Analysis

Users in the minimally invasive treatment groups ceased to mention nearly all of the symptoms being studied significantly earlier versus the abdominal group, even in cases where proportionally more users mentioned the symptom. For example, users in the LH group ceased to mention bleeding at a mean interquartile difference of 1.66 days sooner (*P*=.003) than in AH group. In the VH group, users ceased to mention pain keywords at a mean interquartile difference of 4.00 days sooner (*P*<.001) than in the AH group. Table 2 lists the mean interquartile differences in days between the timing of cessation of each symptom mention by treatment group compared with the AH cohort.

**Table 2:** User symptom keyword survival mean interquartile differences versus abdominal group, in days.

Symptom keyword	Laparoscopic	Vaginal
Family	−9.3^a,b^	−5.0^a,c^
Drugs	−3.6^a,c^	−1.3^a^
Urinary	−7.6^a,b^	−4.6^a,b^
Hormones and emotions	−5.6^a,b^	−5.6^a,b^
Intimacy	−0.3^a^	1^d^
Sleep and fatigue	−6.3^a,b^	−3.6^a,c^
Swelling	−7.0^a,b^	−4.0^a,c^
Pain	−7.0^a,b^	−4.0^a,b^
Fever and infection	−7.3^a,b^	−6.0^a,b^
Digestion	−4.0^a,b^	−2.3^a,b^
Odd sensations	−4.3^a,b^	−2.6^a,c^
Bleeding	−1.6^a,c^	−2.0^a,b^

^a^Values indicate an earlier cessation of mentions.

^b^*P*<.001.

^c^*P*<.01.

^d^Values indicate later cessation of mentions.

## Discussion

### Results Analysis

The HysterSisters forum dataset provides an opportunity not only to broadly sample patient online conversations regarding hysterectomy recovery, benchmarked by date, and type of procedure but also, more broadly, a method by which online conversations can be used to inform perioperative care for similar communities surrounding different clinical experiences.

This analysis provides rich insight into the hysterectomy recovery experience. First, the temporal dynamics of individual engagement on the forum are quite varied. Individuals seek engagement most heavily in the first 3 weeks after hysterectomy. However, there are also specific windows during the recovery in which engagement is desired. For hysterectomy, these peaks in conversation volume occur at 1, 3, and 6 weeks postoperative. In addition, the topic of interest changes as well. During the first week, digestion issues are of considerable concern, but at 1 week and beyond, a relative increase in the percentage of *bleeding* mentions suggests bleeding as a potential focus of assessment, reassurance, or counseling for patients. Conversations related to *intimacy* arise starting at 3 weeks, spiking at 6 weeks postoperatively, coinciding with providers’ *approval* to return to sexual activity; however, this spike may also suggest the presence of a persistent information gap patients seek to fill. That the distribution of keyword mentions remains otherwise constant throughout the 12-week recovery indicates users do continue to desire engagement on all these topics throughout and beyond the standard recovery period. Investigators pursuing similar avenues of research should consider such dynamism when analyzing posting behavior.

Second, procedural variations should be accounted for as they may impact the clinical applications of the research. In our case, the type of hysterectomy was captured as structured data. Individuals undergoing VH make proportionally more mentions of urinary symptoms. This difference in mentions may be because of more women in this cohort undergoing concomitant prolapse or incontinence surgeries, which this analysis does not explore. Clearly, however, patients seek engagement here, and addressing urinary function can help maximize patient satisfaction with their recovery experience. We also noted topics about bleeding occurred more frequently from individuals undergoing the various laparoscopic hysterectomies. This difference in conversation frequency between approaches was in contrast to clinical data presented in the Cochrane review on surgical approach to hysterectomy where no evidence of a difference in the number of individuals with substantial bleeding between laparoscopic and AH groups was seen [22]. Therefore, we undertook a manual investigation into the posts. A 3-gram analysis of the laparoscopic cohort noted *at weeks post* is mentioned significantly more (*P*<.001), and examples include “Vaginal discharge at 10 weeks post hysterectomy,” “New slight spotting at 10 weeks post hysterectomy,” and “Spotting and slight pain at 10 weeks post hysterectomy.” These examples suggest patients may experience resumption of bleeding after a perceived recovery. Although dissolution of delayed absorbable suture is a ready explanation in this instance, the example demonstrates how this type of research reveals opportunities for anticipatory guidance.

Notably, although there are no significant differences in the frequency of mentions of pain-related symptoms, the survival analysis shows the last mention of pain occurring about 7 days earlier for the laparoscopic cohort with about half of these users ceasing to mention pain by postoperative day 20. It is tempting to interpret this finding as half of the users stop experiencing pain at 3 weeks, but this may not be the case. More appropriately, as users’ experience begins to match their expectations for pain or any particular symptom, the need to engage socially may diminish. As noted above, reengagement can occur when expectation-experience mismatching occurs.

Nevertheless, with the exception of intimacy-related keywords, cessation of symptom mentions occurs earlier in the vaginal and laparoscopic cohorts across all symptom groups. Therefore, although our analysis is not intended to deliver concrete recommendations as to the route of hysterectomy, the findings do parallel those in clinical gynecology literature where return to normal activities was found to occur earlier after vaginal and laparoscopic hysterectomies versus the abdominal approach [22].

Taken together, these findings can help guide clinical postoperative care. For example, the interquartile difference in days for cessation of bleeding mentions is only 2.0 days for the vaginal compared with AH groups; therefore, practically, bleeding should be discussed regardless of hysterectomy type and remains a concern throughout the recovery process. In addition, gastrointestinal and genitourinary symptoms should receive focus early (at discharge), whereas providers should be sure to address intimacy issues at the final postoperative visit and reassure patients of their ongoing availability for care as the patients’ needs may continue past the typical 6-week clinical recovery period. We present a simple reminder chart to alert providers to review these critical topics (Textbox 2).

### Conversational Perspectives

Although internet search and online forum usage are rising among patients, patients still overwhelmingly turn to their doctor for medical expertise. In 1 study, 91% of patients sought their doctor for medical diagnosis. However, when asked about *practical advice for coping with day-to-day health situations*, patients were more divided with 43% choosing their doctor and 46% choosing the group including fellow patients, friends, and family [28].

This analysis has important *a priori* limitations. First, symptoms keywords were sorted into taxonomies by a subjective iterative process because the authors must ultimately assign any keyword to a symptom group. The taxonomy may be incomplete because of sparseness of typographical error, unanticipated slang, or proper medical terminology reported unassociated with a symptom (eg, *catheter*). Our taxonomy is therefore included as an appendix.

Second, this analysis should not be seen as equating mentions of symptoms with patient complaint. Deeper analysis could begin to explore subject headers, post content, and conversational patterns related to motivation.

Finally, almost 60% of posts in the Hysterectomy Recovery board did not fall into our taxonomy. Excluded subject headers include a variety of content, including nonsymptom issues (eg, return to activity, comorbid condition issues, or nonmedical topics such as *makeup*), progress updates, or *chatter*.

### Future Directions

This study presents a first look into text analytics to explore the patient experience in gynecologic care and how such research might be conducted in other fields. Focus is limited, however, to the subject headers of posts by site users. Future research analyzing the post body itself and other conversational elements and patterns, we can begin to ascertain the underlying motivation for posting.

**Clinician’s quick guide to hysterectomy postoperative counseling.**

**At discharge**

Discuss: gastrointestinal and genitourinary function; typical delay in return of normal bowel function; symptoms of urinary retention versus return of normal voiding; proper pain medication use; bleeding expectations

**At 1 week**

Discuss: reassure that intermittent light bleeding is normal if present; ensure adequate return of bowel and bladder function; readdress pain control

**At 3 weeks**

Discuss: general check-in with patient; identify individual issues; address hormones, emotional changes, and coping strategies

**At 6 weeks**

Discuss: return to normal activity; return to sexual activity; address hormonal changes if persistent or indicated; review possibility of light bleeding 10 weeks postoperative

Our results demonstrate a timeline of posts with shifting conversational volume in specific areas, with the majority of posts occurring in the first 2 weeks. Future research can integrate this information into existing personalized electronic health programs for recovery from gynecologic surgery to deliver *just-in-time* information to patients [29]. *Push* notifications have been used clinically for such active engagement [28,30,31].

We hope that our results provide insight to both the gynecologic surgeon as to what their patients are discussing after hysterectomy and the data scientist using this information to better analyze similar text-based data sets in other fields.

## Appendix

Multimedia Appendix 1 Symptom keyword taxonomy, alphabetical.

Multimedia Appendix 2 Symptom keyword taxonomy, by mentions.

Multimedia Appendix 3 Patients who mention a symptom during the recovery period. Each patient is counted at most once per symptom, even if the symptom was mentioned multiple times.

## Abbreviations

AH: abdominal hysterectomy
IRB: Institutional Review Board
LH: laparoscopic hysterectomy
PROM: patient-reported outcome measure
VH: vaginal hysterectomy
