# Supplementary material for: Listening to the HysterSisters: A Retrospective Keyword Frequency Analysis of Conversations About Hysterectomy Recovery
Source: JMIR Perioper Med. 2019 Sep 26;2(2):e10728. doi: 10.2196/10728 (PMC7735658; doi:10.2196/10728)
Supplement: Multimedia Appendix 1 [file periop_v2i2e10728_app1.pdf]

Appendix 1: Symptom Keyword Taxonomy, alphabetical. These are the symptom categories considered in this analysis and the keywords which define those symptoms. A thread is considered "relevant" to a keyword if its subject mentions the keyword, and "relevant" to a symptom if its subject is relevant to any of the symptom's keywords.

Symptom	Keywords
**Bleeding**	bleed, bleedin, bleeding, bleeds, blood, bloody, clot, clots, clotting, discharge, incision, incisional, incisions, silver, spotting, weeping
**Digestion**	bowel, bowell, bowelment, bowels, constipate, constipated, constipation, diaherra, diahhrea, diahorrea, diahrea, diahrrea, digestion, digestive, flatulence, gas, gasous, gassing, gasssssss, gassy, gastric, gastritis, gastrointestinal, gasx, gasy, nausated, nausaus, nausea, nauseaous, nauseas, nauseated, nauseaus, nauseous, nauses, nauseus, nausia, nausiated, nausious, poo, pooh, poohing, pooled, pools, poop, pooped, pooping, queasiness, queasy, stool, stools, tummyache, vomit, vomited, vomiting, vomitting
**Drugs**	antibiotic, antibiotics, medication, medications, medicinal, medicine, meds, oxycodone, pill, pills
**Family**	child, childcare, children, daughter, daughters, families, family, husband, husbands, husbavd, kids, son
**Fever & Infection**	degree, fever, feverish, fevers, infected, infection, infections, temp, temperature
**Hormones & Emotions**	angry, cold, colder, cry, crybaby, cryin, crying, cryng, depressed, depression, elestrin, emotion, emotiona, emotional, emotionally, emotionaly, emotionless, emotions, estriadol, estrogel, estrogen, flash, flashed, flashes, flashing, hormone, hormones, hot, hotflash, hotflashes, hotter, irritable, mad, maddening, madness,

	menstrual, mood, moodiness, moods, moody, pms, progesterone, sad, sadder, saddle, sadist, sadness, weepies, weepy
**Intimacy**	intercourse, intimacy, intimancy, intimate, libido, orgasm, sex, sexual, sexuality, sexually, testosterone, testostorone
**Odd Sensations**	dizziness, dizzy, numb, numbness, pinch, pinched, pinches, pinching, pinchy, pressure, pull, pulled, pulling, pulls, tight, tighten, tightening, tighter, tightly, tightness, tingle, tingling, tingly, tugging, woozey, wooziness, woozy
**Pain**	ache, aches, achey, burn, burned, burning, burns, burnung, cramping, cramps, crampy, discomfort, dull, headache, headaches, hurt, hurtful, hurtin, hurting, hurts, itch, itches, itchhhhhhhhh, itchies, itchiness, itching, itchinng, itchy, pain, painful, painfull, painfully, painless, pains, searing, sharp, shooting, sore, soreness, sorer, sores, sorest, stabbing, sting, stinging, stings, tender, tenderness, throb, throbbing
**Sleep & Fatigue**	ambien, awake, awaken, awakened, awakenings, dozing, energy, exhausted, exhausting, exhaustion, exhausts, fatigue, fatigued, insomnia, insomniacs, lethargic, melatonin, nap, napping, naps, resting, restless, sleep, sleeper, sleepers, sleepiness, sleeping, sleepless, sleeplessness, sleepling, sleeps, sleepy, slept, tire, tired, tiredd, tiredness
**Swelling**	bloat, bloated, bloating, bloaty, pooching, poochy, poof, poofy, swell, swelled, swellies, swelling, swellings, swellinng, swellly, swells, swelly, swellybelly, swellybellyband, swellyy, swollen
**Urinary**	bladder, bladders, incontinance, incontinence, incontinent, leak, leakage, leakages, leaked, leaking, leaks, leaky, pee, pee'ing, peed, peeing, peepee, urinary, urinate, urinatinating, urinating, urination, urine, uti, utis, void
