# Supplementary material for: Listening to the HysterSisters: A Retrospective Keyword Frequency Analysis of Conversations About Hysterectomy Recovery
Source: JMIR Perioper Med. 2019 Sep 26;2(2):e10728. doi: 10.2196/10728 (PMC7735658; doi:10.2196/10728)
Supplement: Multimedia Appendix 2 [file periop_v2i2e10728_app2.pdf]

Appendix 2: Symptom Keyword Taxonomy, by mentions. The numbers in the Symptom column indicate the total number of threads relevant to each symptom. The numbers in parenthesis indicate the total number of threads relevant to each keyword.

**Symptom**	**Keywords**
**Pain: 12474**	pain (7471), sore (654), pains (618), burning (455), cramping (429), painful (375), cramps (337), sharp (299), headaches (270), itching (267), hurt (257), itchy (253), discomfort (200), hurts (195), hurting (193), headache (141), soreness (114), tender (112), aches (111), stabbing (106), tenderness (106), ache (93), crampy (67), stinging (67), shooting (57), throbbing (36), dull (34), itch (33), burn (27), achey (19), burns (13), sores (11), itches (10), itchiness (9), searing (9), sting (8), burned (5), itchies (5), hurtful (4), painfull (4), painless (4), stings (3), painfully (2), burnung (1), hurtin (1), itchhhhhhhhh (1), itchinng (1), sorer (1), sorest (1), throb (1)
**Bleeding: 7491**	bleeding (2532), incision (1906), discharge (1161), spotting (924), blood (677), incisions (220), silver (160), clot (144), clots (127), bleed (78), bloody (31), incisional (26), clotting (19), weeping (16), bleeds (8), bleedin (3)
**Hormones & Emotions: 3643**	hot (781), flashes (523), emotional (414), hormones (275), crying (254), cold (216), depression (212), pms (206), depressed (195), hormone (178), sad (157), estrogen (135), mood (133), emotions (132), flash (80), cry (70), angry (64), weepy (62), mad (41), emotionally (40), menstrual (34), moody (32), progesterone (29), irritable (23), sadness (20), hotflashes (17), moods (12), moodiness (10), saddle (9), estrogel (8), emotion (6), flashing (6), hotflash (5), madness (5), elestrin (4), cryin (3), flashed (3), emotionless (2), weepies (2),

	colder (1), crybaby (1), cryng (1), emotiona (1), emotionaly (1), estriadol (1), hotter (1), maddening (1), sadder (1), sadist (1)
**Digestion: 2872**	gas (867), bowel (624), nausea (393), constipation (332), bowels (148), stool (98), poop (83), nauseous (66), constipated (62), vomiting (60), gassy (37), nauseated (30), pooping (22), stools (22), poo (19), digestive (18), pooped (12), vomitting (11), digestion (9), flatulence (7), bowell (5), queasy (5), vomited (4), diahhrea (3), diahrea (3), gastric (3), gastrointestinal (3), gasx (3), nauseas (3), nauseus (3), pooh (3), queasiness (3), constipate (2), gasy (2), nausated (2), nauseaus (2), pooled (2), vomit (2), bowelment (1), diaherra (1), diahorrea (1), diahrrea (1), gasous (1), gassing (1), gasssssss (1), gastritis (1), nausaus (1), nauseaous (1), nauses (1), nausia (1), nausiated (1), nausious (1), poohing (1), pools (1), tummyache (1)
**Sleep & Fatigue: 2694**	tired (586), sleep (525), sleeping (373), insomnia (356), fatigue (194), exhausted (153), energy (128), exhaustion (66), restless (64), tiredness (47), resting (33), sleepless (32), sleepy (31), slept (30), fatigued (29), awake (28), ambien (17), nap (10), melatonin (8), sleeper (8), sleepers (8), tire (8), sleeplessness (6), napping (5), naps (5), awaken (2), awakened (2), exhausting (2), insomniacs (2), sleepiness (2), awakenings (1), dozing (1), exhausts (1), lethargic (1), sleepling (1), sleeps (1), 2ired (1)
**Urinary: 2647**	bladder (1374), uti (266), urine (208), pee (162), leaking (149), urination (138), urinating (132), peeing (99), leakage (90), urinary (88), incontinence (58), urinate (56), leaky (14), peed (9), leak (7), utis (7), void (5), leaks (4), leaked (3), incontinance (2), incontinent (2), pee'ing (2), bladders (1), leakages (1), peepee (1), urinatinating (1)

**Intimacy: 2110**	sex (1386), intercourse (216), intimacy (198), orgasm (110), libido (82), sexual (81), intimate (30), testosterone (12), sexually (8), sexuality (3), intimancy (1), testostorone (1)
**Swelling: 2009**	swelly (819), swelling (485), swollen (438), bloating (122), bloated (97), swell (14), bloat (13), swellybelly (13), swelled (9), swells (5), poof (3), poochy (2), swellly (2), bloaty (1), pooching (1), poofy (1), swellies (1), swellings (1), swellinng (1), swellybellyband (1), swellyy (1)
**Odd Sensations: 1662**	pressure (566), numbness (266), dizzy (175), numb (146), pulling (134), dizziness (96), tingling (65), tight (62), pulled (48), pinching (37), tugging (24), pull (21), tightness (20), tingly (12), pinched (6), tightening (6), pinch (5), tingle (5), pinches (4), woozy (4), tighter (2), pinchy (1), pulls (1), tighten (1), tightly (1), woozey (1), wooziness (1)
**Fever & Infection: 1405**	infection (805), fever (361), infections (71), infected (66), temp (60), temperature (29), fevers (18), feverish (6), degree (2)
**Drugs: 634**	meds (392), pills (69), medication (62), antibiotics (47), medicine (26), pill (21), antibiotic (11), medications (3), oxycodone (3), medicinal (1)
**Family: 486**	husband (136), children (118), kids (100), family (72), child (27), husbands (16), son (10), daughter (9), families (2), childcare (1), daughters (1), husbavd (1)
