# Supplementary figures and images for: Listening to the HysterSisters: A Retrospective Keyword Frequency Analysis of Conversations About Hysterectomy Recovery
Source: JMIR Perioper Med. 2019 Sep 26;2(2):e10728. doi: 10.2196/10728 (PMC7735658; doi:10.2196/10728)

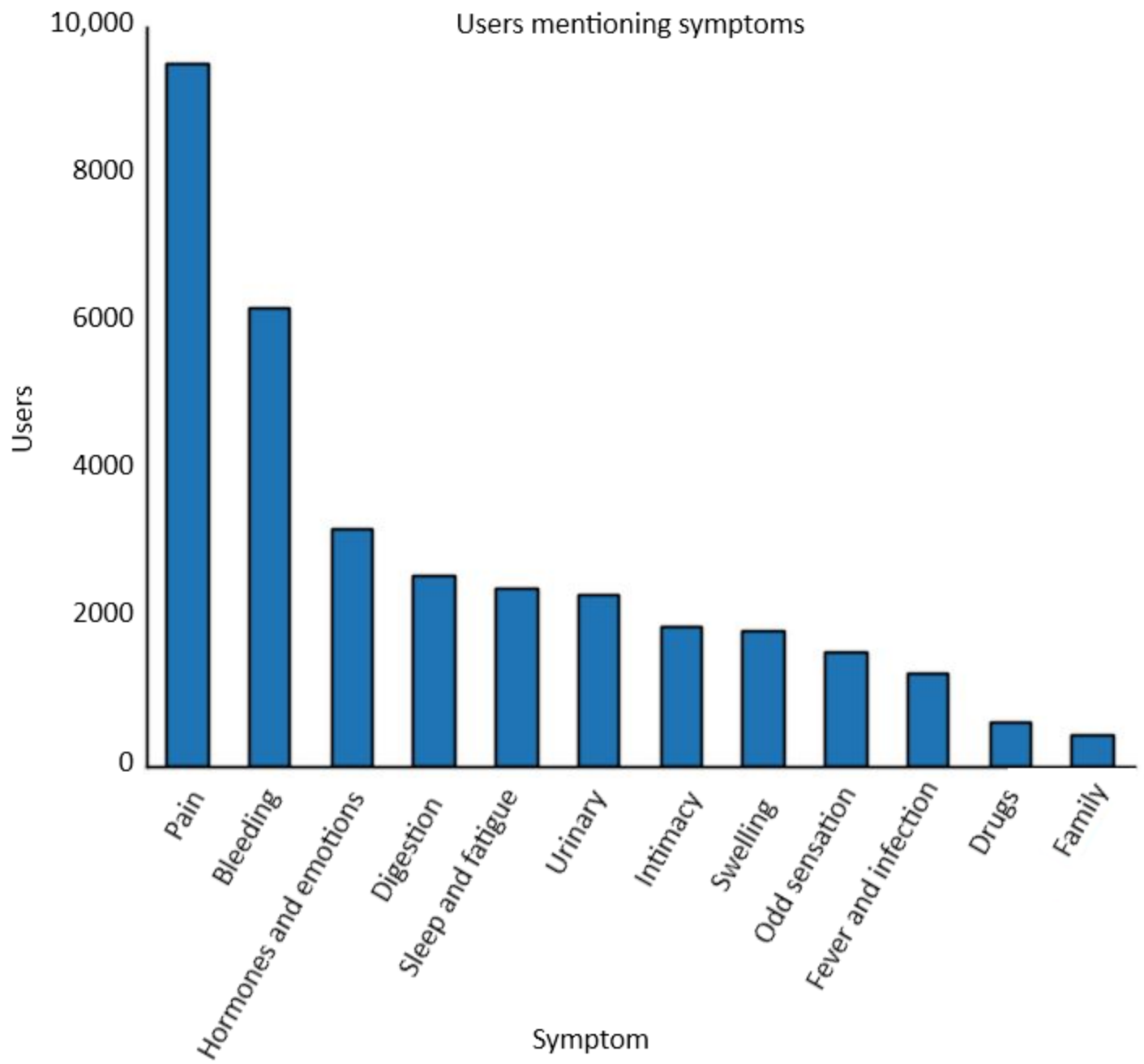

Supplement: Multimedia Appendix 3 [file periop_v2i2e10728_app3.pdf]
